# 4-Hydroxyphenylacetic Acid Prevents Acute APAP-Induced Liver Injury by Increasing Phase II and Antioxidant Enzymes in Mice

**DOI:** 10.3389/fphar.2018.00653

**Published:** 2018-06-19

**Authors:** Hongqiong Zhao, Zhihui Jiang, Xuemei Chang, Huiting Xue, Wumaierjiang Yahefu, Xiaoying Zhang

**Affiliations:** ^1^College of Veterinary Medicine, Xinjiang Agricultural University, Xinjiang, China; ^2^Research Center of Modern Biotechnology, Anyang Institute of Technology, Anyang, China; ^3^College of Veterinary Medicine, Northwest A&F University, Yangling, China; ^4^Department of Biology, Centre of Molecular and Environmental Biology, University of Minho, Braga, Portugal

**Keywords:** polyphenols, 4-hydroxyphenylacetic acid 4-HPA, acetaminophen APAP, hepatotoxicity, oxidative stress, nuclear factor erythroid 2-related factor, Nrf2

## Abstract

Acetaminophen (APAP) overdose is the principal cause of drug-induced acute liver failure. 4-hydroxyphenylacetic acid (4-HPA), a major microbiota-derived metabolite of polyphenols, is involved in the antioxidative action. This study seeks to investigate the ability of 4-HPA to protect against APAP-induced hepatotoxicity, as well as the putative mechanisms involved. Mice were treated with 4-HPA (6, 12, or 25 mg/kg) for 3 days, 1 h after the last administration of 4-HPA, a single dose of APAP was intraperitoneally infused for mice. APAP caused a remarkable increase of oxidative stress markers, peroxynitrite formation, and fewer activated phase II enzymes. 4-HPA increased Nrf2 translocation to the nucleus and enhanced the activity of phase II and antioxidant enzymes, and could thereby ameliorate APAP-induced liver injury. Studies reveal that 4-HPA, as an active area of bioactive dietary constituents, could protect the liver against APAP-induced injury, implying that 4-HPA could be a new promising strategy and natural hepatoprotective drug.

## Introduction

Acetaminophen (*N*-acetyl-p-aminophenol, APAP) is a common and effective drug for analgesic and antipyretic uses. When consumed in overdose quantities, it has high toxicity and leads to liver failure and acute liver failure (ALF) (Larson et al., 2005). In the United States and the United Kingdom, high dosing on APAP has been reported as the main reason for drug-induced liver injury (Blieden et al., 2014). In China, 0.40% drug induced liver injury patients were induced by APAP (Zhou et al., 2013). APAP undergoes Phase I and II detoxification pathways in the liver; about 85% of APAP is recovered as APAP-gluc and APAP-sulf by hepatic phase II conjugation enzymes, including UDP-glucuronosyltransferases (UGTs) and sulfotransferases (SULTs), and excreted in the urine. It is widely accepted that the highly reactive intermediate of *N*-acetyl-p-benzoquinone imine (NAPQI) is metabolized through hepatic phase I reactions by the cytochrome P-450 (CYP) pathway (primarily by CYP2E1). In the case of an overdose of APAP, the phase II reaction becomes saturated and the over-production of NAPQI depletes liver glutathione (GSH) and modifies cellular proteins. This disturbs redox balance and leads to mitochondrial damage and centrilobular necrosis, which leads to oxidative stress-induced liver injury (James et al., 2003; Xie et al., 2015).

It is known that polyphenols, a wealth of secondary plant metabolites in edible plants, is attributed to a broad spectrum of bene?cial pharmacological activities in humans (Li et al., 2014). Evidence indicates that polyphenols were less absorbed in the small intestine but were accumulated in the colon (Cardona et al., 2013). A large number of low-molecular-weight phenolic acids, derived from polyphenols by colonic microflora, could be absorbed into systemic circulation (Selma et al., 2009; Prior et al., 2010) and result in bioavailability. Previous publications have investigated 4-hydroxyphenylacetic acid (4-HPA) as the major metabolite of proanthocyanidins, kaempferol (Deprez et al., 2000; Moradi-Afrapoli et al., 2016). 4-HPA possesses anxiolytic (Vissiennon et al., 2012) and antiplatelet properties (Kim et al., 1998). In our preliminary work, we investigated the ability of microbiota-derived metabolites 3,4-HPA of quercetin to attenuate APAP-induced liver injury in mice (Xue et al., 2016). These ?ndings suggested that metabolites produced from polyphenols by intestinal micro?ora might be hepatoprotection of pharmacologically active compounds. 4-HPA is one of the major metabolites in polyphenols (Zabela et al., 2016). Molecular docking study revealed that the position of the hydroxyl group in 3,4-HPA and 4-HPA have different binding sites on CYP2E1. The interaction of 4-HPA was comparatively higher than the 3,4- HPA. 4-HPA docking scores were par with 3,4-HPA acid. It’s comparatively higher than the standard drug *N*-acetylcysteine (Supplementary Material), which indicates the most comfortable position of ligand in a receptor, compared to both their docking energy against the Nrf2 and CYP2E1. The results indicate that 4- hydroxyphenylacetic acid has potential to be developed as hepatocellular carcinoma (HCC) therapy.

## Materials and Methods

### Experimental Setting

Mice were randomly assigned to six groups. The control group (Control) and negative prevent group (APAP) were administrated 0.9% saline solution and 300 mg/kg APAP (Xie et al., 2016), respectively. Clinically, APAP-induced hepatotoxicity may be attenuated by *N*-Acetyl-L-cysteine (NAC) replenishing the GSH store and enhancing hepatic recovery. However, there are limits to NAC therapy, such as the narrow therapeutic time window for NAC administration (delayed treatment may lead to liver injury) (Corcoran and Wong, 1986; Delanty and Fitzgerald, 1996). NAC could not decrease the hepatotoxicity at a dose of 25 mg/kg compared to a dose of 100 mg/kg in preliminary experiments. In this study, the positive prevent group and experimental groups were treated with *N*-acetylcysteine (NAC100, 100 mg/kg per day) (Nelson et al., 2015) or 4-HPA (4HPA6, 4HPA12, 4HPA25, 6, 12, 25 mg/kg per day) (Sigma, St. Louis, MO, United States), respectively, by intragastric gavage for three consecutive days. One hour after the last administration of 4-HPA, NAC, or saline solution, a single dose of 300 mg/kg APAP was intraperitoneally infused for mice in the negative control group, positive control group, and experimental groups. The drugs were dissolved in 0.9% saline solution immediately before conducting the experiment. Twenty-four hours after APAP was infused, the mice were euthanized and harvested for blood and liver tissue samples. The liver was fixed in 4% para-formaldehyde for histological analysis. Remaining liver tissue was stored in a deep freezer at -80°C for further biochemical analysis.

### Serum ALT and AST Assays

Blood samples were centrifuged at 4°C for 10 min at 2,500 ×*g* to separate the serum. Alanine aminotransferase (ALT) and aspartate aminotransferase (AST) enzymatic activities of the serum were measured with commercial diagnostic kits (Nanjing Jiancheng Institute of Biotechnology, Nanjing, China).

### Measurement of Biochemical Index of GSH, SOD, CAT, GPx, and MDA

Frozen liver tissues were homogenized in 4°C PBS and the homogenate was centrifuged at 2,500 ×*g* at 4°C for 10 min. The supernatants were obtained and assayed for malondialdehyde (MDA), superoxide dismutase (SOD), GSH peroxidase (GPx), catalase (CAT), and GSH levels using commercially available assay kits according to the manufacturer’s instructions (Nanjing Jiancheng Bioengineering Institute, Nanjing, China). Tissue homogenate was analyzed by protein Bradford assay (Tiangen Biotech, Beijing, China).

### Hepatic UGTs, SULTs, and GST Levels Measurement

Hepatic profile of UGTs, SULTs, and GST was analyzed using ELISA kits (Hufeng Biotechnology, Shanghai, China).

### Real-Time qPCR Assays

The total mRNA was extracted from the 100 mg of tissue derived from the lobe of liver sample using a commercially available kit (Tiangen Biotech, Beijing, China) according to the manufacturer’s instructions, and reverse-transcribed into cDNA using HiScript^TM^ RT SuperMix for qPCR (Vazyme Biotech, Nanjing, China) with a Veriti Thermal Cycler (Applied Biosystems, Waltham, United States). Oligonucleotide primers are listed in **Table 1**. Real-time qPCR was performed on an iQ5 Real-Time PCR Detection System (Bio-Rad, United States) with SYBR^®^ Green Master Mix (Vazyme Biotech, Nanjing, China). The relative expression of mRNA was expressed by 2^-(ΔΔCt)^ and normalized to β-actin, an internal control gene.

**Table 1:** Primers Used for Real-Time PCR.

Target	Forward	Reverse
UGT1A1	CACGCTGGGAGGCTGTTAGT	CACAGTGGGCACAGTCAGGTA
UGT1A6	CACGTGCTACCTAGAGGCACAG	GACCACCAGCAGCTTGTCAC
UGT1A9	GAAGAACATGCATTTTGCTCCT	CTGGGCTAAAGAGGTCTGTCATAGTC
SULT1A1	CCCGTCTATGCCCGGATAC	GGGCTGGTGTCTCTTTCAGAGT
SULT2A1	TAGGGAAAAATTTAGGGCCAGAT	TTGTTTTCTTTCATGGCTTGGA
GSTa1	CCGTTACTTGCCTGCCTTTG	TGCAGCTTCACTGAATCTTGAAAG
GSTm1	ATGGTTGTCCAGGTCTGG	CGCCATCTTGTGCTACATT
CYP2E1	CACCGTTGCCTTGCTTGTCTG	CTCATGAGCTCCAGACACTTC
Nrf2	ACACGGTCCACAGCTCATCAT	TTGGCTTCTGGACTTGGAAC
β-actin	TATTGGCAACGAGCGGTTCC	GGCATAGAGGTCTTTACGGATGTC

### Western Blot Analysis

Glutamate-cysteine ligase catalytic (GCLC), 3-Nitrotyrosine (3-NT), Nrf2, and CYP2E1 were detected using total liver protein, cytosolic and nuclear extracts, and microsome, respectively.

The total liver protein was extracted with 4°C NP-40 lysis buffer (Beyotime Institute of Biotechnology, Beijing, China) containing 1 mM PMSF. Cytosolic and nuclear extracts were prepared with a commercially available kit according to the manufacturer’s instructions (Vazyme Biotech, Nanjing, China).

For the microsomal preparation, the liver homogenate was centrifuged at 9,000 × *g* for 20 min at 4°C, and the supernatant was transferred to a tube with 88 mM CaCl_2_ at a ratio of 0.4 mL per 1 g starting liver tissue; after being shaken on ice for 5 min, the mixture was centrifuged at 27,000 ×*g* for 20 min. The pellet was collected and resuspended in 50 mM Tris–HCl mixed with 20% glycerol.

The protein concentration was determined by BCA assay kit (Beyotime Institute of Biotechnology, Beijing, China). Equal amounts of extracted protein (50 μg) were separated on 12% SDS-PAGE, proteins were transferred to a PVDF membrane, and blocked with TBST containing 5% non-fat milk for 2 h at room temperature. Subsequently, the membranes were incubated overnight at 4°C with primary antibodies which were 3-NT (dilution 1:1 000. Abcam, Cambridge, United Kingdom). GCLC (dilution 1:1 000. Abcam, CA, United Kingdom) CYP2E1 (dilution 1:1 000. Tiangen Biotech, Beijing, China), Nrf-2 (dilution 1:500. Santa Cruz Biotechnology, CA, United States), Lamin B (dilution 1:500. Santa Cruz Biotechnology, CA, United States), and GAPDH (dilution 1:1000. Tianjin Sungene Biotech, Tianjin, China), and incubated with secondary antibodies (dilution 1:2 000, Thermo Fisher Scientific, California, United States) for 2 h at room temperature. Before analyzing the fold change to control ratio, images of the blots were visualized by Western Bright ECL (Advansta, California, United States).

### Histological Analysis

The liver tissues were fixed with 4% paraformaldehyde and embedded into paraffin. Through deparaffinization and dehydration, the liver samples were sectioned 5 μm and stained with hematoxylin and eosin (H&E). Then, the periportal areas were captured by bright field light microscopy (Olympus, Tokyo, Japan). Five fields in each mouse were randomly chosen. The histological analysis showed massive necrosis, centrilobular ballooning degeneration, sinusoidal congestion, and lymphocyte infiltration of liver lesions.

### Statistical Analysis

All the experimental data were expressed as mean ± SD. The significant differences from the respective controls in all experiments were assessed by one-way ANOVA using SPSS (version 20.0, IBM Corporation, United States).

## Results

### 4 HPA-Attenuated APAP-Induced Hepatotoxicity

**FIGURE 1:**
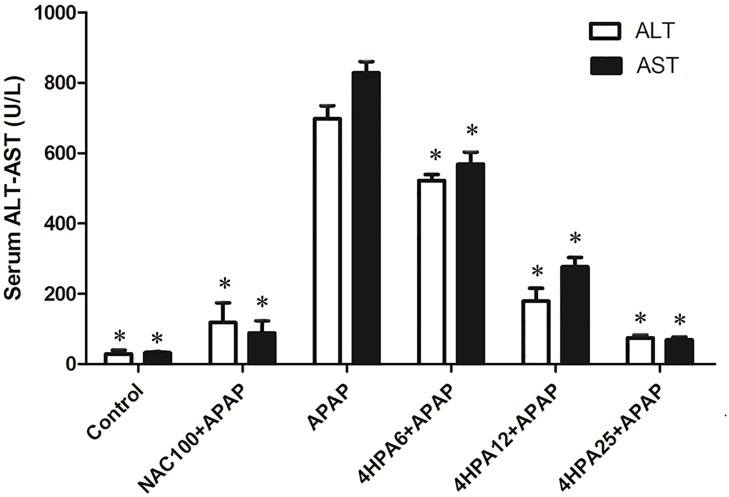
Effects of 4-HPA on APAP-induced increase in serum transaminases levels. Each value represents the mean ± SD of eight animals per group. ^∗^ indicates statistically different group with APAP group (*p* < 0.05; AST and ALT, respectively). (1) Control: control group; (2) NAC100+APAP: treated with 100 mg/kg per day NAC and 300 mg/kg APAP; (3) APAP: a single dose of 300 mg/kg APAP; (4) 4HPA6+APAP: treated with 6 mg/kg per day 4-HPA and 300 mg/kg APAP; (5) 4HPA12+APAP: treated with 12 mg/kg per day 4-HPA and 300 mg/kg APAP; (6) 4HPA25+APAP: treated with 25 mg/kg per day 4-HPA and 300 mg/kg APAP.

As shown in **Figure 1**, the administration of APAP dramatically elevated the serum ALT and AST activities levels compared with the control group. In contrast, in the groups pretreated with 4-HPA, ALT and AST activities were able to prevent APAP-induced liver injury. The increase in serum ALT and AST values induced by APAP was also prevented in the NAC+APAP group.

### 4 HPA-Alleviated APAP-Induced Histopathological Changes in the Liver

**FIGURE 2:**
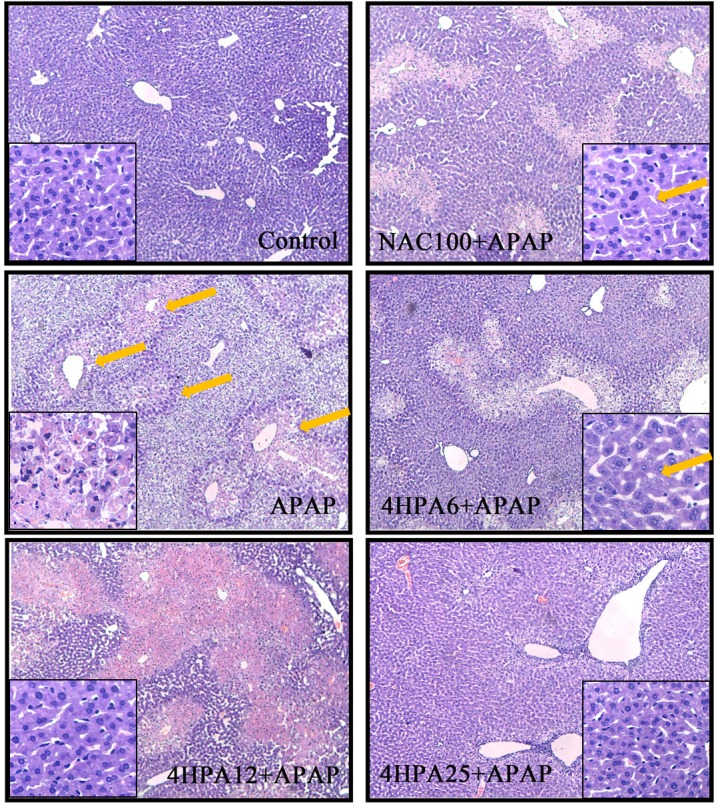
Effects of 4-HPA on hepatic histopathological alterations following APAP treatment. Typical images were chosen from each experimental group. Original magnification was 10 × 10 and 40 × 10. The arrows mean liver injury.

As shown in **Figure 2**, normal hepatocytes were arranged in plates around a central vein and separated by blood sinusoids. APAP caused hepatocellular injury, markedly massive necrosis, centrilobular ballooning degeneration, sinusoidal congestion, and lymphocyte infiltration (marked with arrows). Liver congestion and centrilobular necrosis were decreased in a dose-dependent manner by 4-HPA pretreatment. Distinctly, the groups pre-treated with 25 mg/kg 4-HPA were unscathed and appeared almost normal, with just a few swollen hepatocytes observed in the centrilobular zone. A small number of hepatocytes in the centrilobular region exhibited cytoplasmic vacuolation in the 100 mg/kg NAC pretreated group (marked with arrows).

### 4-HPA Inhibited APAP-Induced Oxidative Liver Stress

**FIGURE 3:**
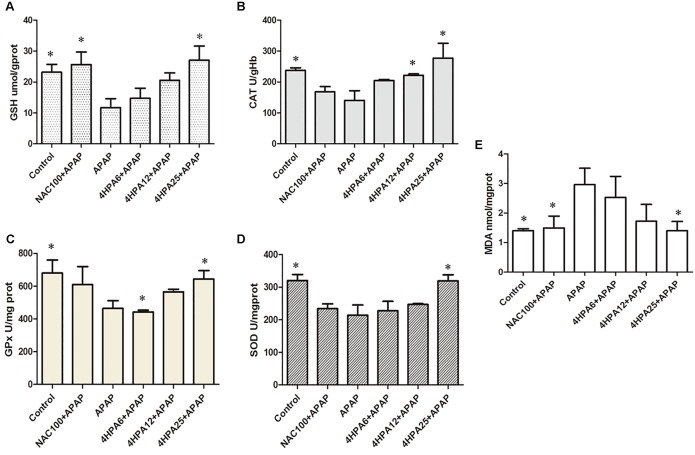
Effects of 4-HPA on oxidative stress induced by APAP. **(A)** GSH, **(B)** CAT, **(C)** GPx, **(D)** SOD **(E)** MDA. Each value represents the mean ± SD of eight animals per group. ^∗^ Above the bars indicate a statistically different group with APAP group (*p* < 0.05).

The injection of APAP resulted in significantly increased levels of MDA and depleted GSH levels when compared to the control, indicating APAP-induced oxidative liver stress. APAP also decreased the GPx, CAT, and SOD activity levels by 68%, 59%, and 67%, respectively. Administration of NAC prevented the collapse of the redox balance caused by APAP hepatotoxicity. In addition, 4-HPA pretreatment significantly prevented all the effects of APAP in a dose-dependent manner (**Figure 3**). Relative to the NAC group, 25 mg/kg 4-HPA pre-treatment was induced to prevent the CAT and SOD levels.

### 4-HPA Inhibited APAP-Induced Nitrotyrosine Adduction

**FIGURE 4:**
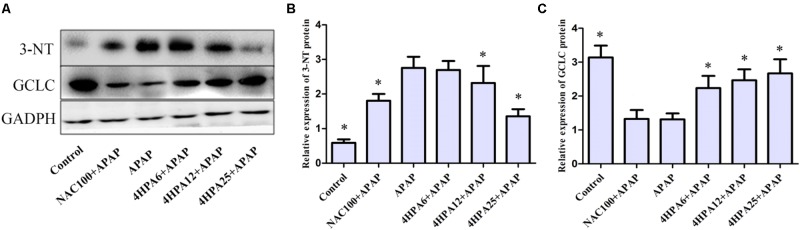
Effects of 4-HPA on protein expression of 3-NT and GCLC following APAP overdose. **(A)** Representative blots of 3-NT, GCLC, and GAPDH expression. Quantification of 3-NT. **(B)** GCLC **(C)** protein levels. ^∗^ Above the bars indicate statistically a different group with APAP group (*p* < 0.05). Each value represents the mean ± SD of eight animals per group.

3-Nitrotyrosine formation was 600% higher in APAP groups compared to the control group (**Figure 4B**). Pre-administration of 4-HPA at doses of 6, 12, and 25 mg/kg decreased 3-NT adduction by 32%, 50%, and 68%, respectively, as compared to APAP groups. 4-HPA was less effective at increasing GCLC expression, observed in a dose-dependent manner.

### 4-HPA Suppressed the Expression of CYP2E1

**FIGURE 5:**
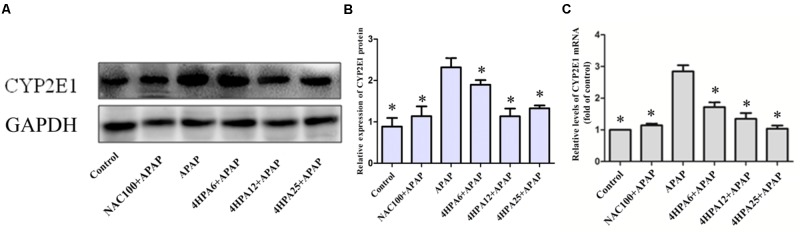
Effects of 4-HPA on protein and mRNA expression of CYP2E1 following APAP overdose. **(A)** Representative blots of CYP2E1 and GAPDH expression. **(B)** Quantification of CYP2E1 protein levels. **(C)** Relative mRNA levels of CYP2E1. Each value represents the mean ± SD of eight animals per group. ^∗^ Above the bars indicate a statistically different group with APAP group (*p* < 0.05).

According to the data of protein expression and mRNA levels of CYP2E1 using WB and qPCR, we observed APAP alone enhanced the levels of CYP2E1 mRNA and protein levels. Meanwhile, with dose-dependent 4-HPA, CYP2E1 was significantly suppressed (**Figure 5**).

### 4-HPA Enhanced the Protein and mRNA Levels of Phase II Enzymes

**FIGURE 6:**
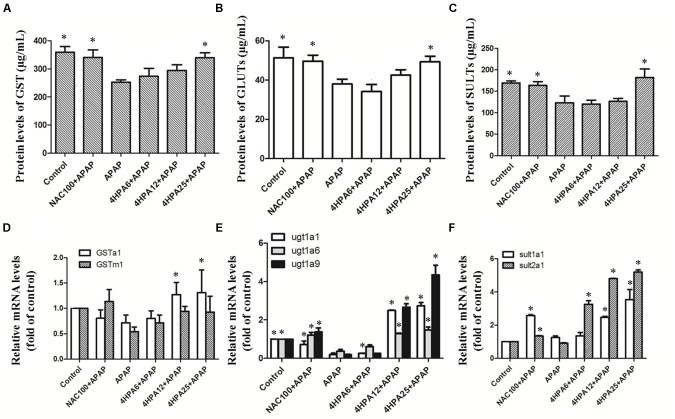
Effects of 4-HPA on mRNA and levels of phase II enzymes following APAP overdose. The levels of GST **(A)**, UGTs **(B)**, and SULTs **(C)** were determined by ELISA, and the mRNA levels of GSTa1 and GSTm1 **(D)**, UGT1A1, UGT1A6 and UGT1A9 **(E)**, SULT1A1 and SULT2A1 **(F)** were determined by real time PCR. Each value represents the mean ± SD of eight animals per group. ^∗^ Above the bars indicate a statistically different group with APAP group (*p* < 0.05).

As indicated in **Figure 6**, Phase II enzyme levels were not affected by the lowest dose of 4-HPA. SULT levels were mild fluctuations in the middle dose of 4-HPA. Contextually, compared to APAP alone, the highest dose of 4-HPA pretreatment caused 25% (GST), 30% (SULT), and 23% (GLUT) up-regulation. At the same time, similar levels were observed between the positive control group of NAC+APAP and the 4-HPA25+APAP groups. Overall, 4-HPA could induce phase II enzyme growth activities in APAP-induced damage.

A single dose of APAP significantly down-regulated the hepatic mRNA activities of phase II enzymes versus the control group. Likewise, 4-HPA pretreatment normalized the mRNA levels of phase II enzymes in a dose-dependent manner. The 4-HPA pretreatment at a final dose of 25 mg/kg markedly and selectively up-regulated the target genes of phase II enzymes and resulted in higher up-regulation than that of the control group by 270%, 400%, and 500% or UGT1A1, UGT1A9, and SULT2A1, respectively. The mRNA levels of UGT1A9 showed harsh, dose-dependent fluctuations compared to others in 4-HPA intake groups.

### 4-HPA Induces Expression of Nrf2 and Downstream Gene

**FIGURE 7:**
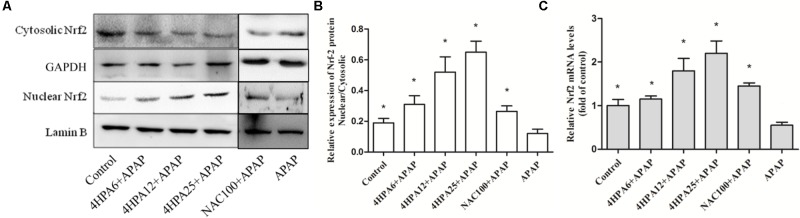
4-HPA induces expression of Nrf2. **(A)** Western blots of Nrf2, Lamin B, and GAPDH expression. **(B)** Nuclear Nrf2/Cytosolic Nrf2 content ratios and the densities of nuclear and cytosolic Nrf2 normalized by Lamin B and GAPDH. **(C)** Relative mRNA levels of Nrf2. Each value represents the mean ± SD of eight animals per group. ^∗^ Above the bars indicate a statistically different group with the APAP group (*p* < 0.05).

Nrf2 expression in the livers of mice was examined using Western blotting and real time-PCR. The protein levels of nuclear Nrf2 were increased by 170% and 230% in pre-treated 12 and 25 mg/kg 4-HPA groups, respectively, compared with the control group. Consistent with protein expression, mRNA levels displayed the same trend (**Figure 7**). Nuclear Nrf2 also showed a slight increase in 4-HPA pre-treatment groups. Moreover, compared with APAP alone group, 4-HPA pretreatment groups had a significantly enhanced expression of GCLC (**Figure 4C**), which is the target gene of Nrf2 (**Figure 7C**). The results clearly demonstrate that 4-HPA stimulated the translocation of Nrf2 expression to the nucleus in a dose-dependent manner.

## Discussion

Growing evidence suggests that dietary polyphenols could be a therapy for the prevention of disease and management of health (Mikell and Khan, 2012). For example, polyphenols can regulate the expression of drug induced liver injury through the p38/NF-κB pathway (Liu et al., 2015). However, the polyphenol was poorly absorbed in the gastrointestinal tract (Chen et al., 2005). Colonic bacteria can covert polyphenol into a large number of simple phenolic acids that can be absorbed into the circulation (Prior et al., 2010), and can exert biological effects in the body. 4-HPA is the main metabolite of polyphenols by intestinal microflora Clostridium orbiscinder sp. Nov. (Winter et al., 1991). The human intestinal bacterium of Clostridium orbiscinder sp. Nov is capable of cleaving the C-3-C-4 bond of the quercetin and might be responsible for their pharmacological effects (Griffiths and Smith, 1972; Moradi-Afrapoli et al., 2016). The results show that 3,4-HPA and 4-HPA have different binding sites on CYP2E1 and 3,4-HPA were docked with CYP2E1 in hydroxyl group (Supplementary Figure S1) and 4-HPA docked with CYP2E1 in carboxyl group (Supplementary Figure S2). The interaction of 4-HPA was comparatively higher than the 3,4- HPA. In the present study suggested that 4-HPA may use potential drug candidate for the hepatocellular carcinoma. This study, which primarily focuses on the effect of 4-HPA on acute acetaminophen toxicity in mice, investigates the health implications of metabolic phenolic acids. AST and ALT are secreted into the bloodstream and are sensitive biomarkers used in the evaluation of hepatotoxicity (Waters et al., 2001; Jiang et al., 2016). In this study, we have observed hepatic histopathological lesions and high levels of ALT and AST in the serum of APAP-induced mice compared with the control group. The data show that 4-HPA pretreatment alleviates these alters in a dose-dependent manner, indicating the protective ability of 4-HPA to prevent APAP-induced injury to the liver.

Using the model of APAP-treated mice, hepatic dysfunction is majorly caused by oxidative stress. APAP could be metabolized by CYP2E1 enzymes into NAPQI, which undergoes chemical and enzymatic conjugation to GSH in APAP toxicity (Michael Brown et al., 2012). This could lead to lipid peroxidation, nitrated proteins (Hinson et al., 2000), and subsequent tissue damage. It has been demonstrated that antioxidant enzyme activities could be reduced by APAP overdose, and some treatments have been tested to recover enzymatic function (Carvalho et al., 2013). Here, APAP depleted hepatic GSH, enhanced MDA concentrations, and massively increased 3-NT protein adduction, which are indicators of oxidative stress, along with lowered GPx, SOD, and CAT concentration. The mice that were pretreated with 4-HPA normalized the concentration of antioxidant enzymes and returned MDA levels to normal. The data indicate antioxidant enzymes, especially CAT, as key protective actions induced by 4-HPA against APAP-induced liver injury. It was demonstrated that APAP caused the increase of CYP2E1 expression in the liver. 4-HPA markedly suppressed the levels of CYP2E1 expression and enhanced the antioxidant enzyme levels, which may be due to the powerful antioxidant, suggesting a potential antioxidative effect associated with 4-HPA. UGT, SULT, and GST catalyzed metabolism, making a high contribution to APAP clearance (Ansher et al., 1983; Fakurazi et al., 2012), and then formed urinary acetaminophen mercapturates. In this study, seven kinds of the gene, which are major enzymes and primary isoforms of UGTs, SULTs, and GST, were involved in the detoxification of APAP. This research clarifies that phase II enzyme protein and mRNA expression were increased by 4-HPA in a dose-dependent manner. Meanwhile, 4-HPA markedly enhanced the mRNA levels of UGT1A9 and SULT2A1. The activity of the UGTs, SULTs, GST, and CYP2E1 were not tested in this study. These measures are relevant and should be further assessed to validate one of the hypotheses that 4-HPA attenuates the APAP-induced oxidative stress by the findings of the present study. These results show that the regulation of phase II enzymes is likely to join in the mechanism for functional changes by 4-HPA in this study.

Phase II enzymes are essential for inducing gene expression by Nrf2. The importance of Nrf2 is evident from reports showing that the levels of phase II gene are significantly reduced in Nrf2-deficient mice and that the induction of phase II genes is abolished by the Nrf2 disruption. (Kalthoff et al., 2010; Gum and Cho, 2013). One of the reasons is that ARE is a *cis*-acting DNA response sequence located in the regulatory regions of phase II genes with the consensus of TGAG/CNNNGC (N represents any base). APAP undergoes phase II detoxification pathways in the liver; about 85% of APAP is recovered as APAP-gluc and APAP-sulf by hepatic phase II conjugation enzymes, including UGTs and SULTs. As the result shows, 4-HPA significantly increased the levels of SULTs and UGTs in **Figure 6**. Moreover, some reports have demonstrated that Nrf2-null mice displayed higher susceptibility to APAP-induced liver injury, indicating that the Nrf2 battery serves as a target of hepatoprotection (Okawa et al., 2006; Klaassen and Reisman, 2010). Nrf2, a Cap’n’collar/basic leucine zipper (CNC-bZIP) families of proteins, is regarded as the master regulator of oxidant defense. The pretreatment of mice with 4-HPA induces up-regulation of nuclear Nrf2 expression and mRNA levels in a dose-dependent manner. Lin et al. (2015) found that GCLC expression linked with activation of Nrf2 signaling by knockdown of Nrf2 in HepG2 cells. Furthermore, GCLC, a rate-limiting enzyme, is a major antioxidant molecule in the synthesis of GSH. 4-HPA increases protein expression of GCLC, which is a downstream gene of Nrf2, resulting in enhanced GSH levels. These results suggest that Nrf2 is involved in detoxification and antioxidant signaling pathways induced by 4-HPA.

Previous studies have shown that phenolic acids directly scavenge free radicals or indirectly increase endogenous cellular antioxidant defenses, via the activation of Nrf2 transcription factor pathways (Kelsey et al., 2010). We have determined that 4-HPA is a poor scavenger of free radicals using a common method of the radical 2,2-diphenyl-1-picrylhydrazyl (DPPH) test (data not shown). Combined with the existing results, 4-HPA may have potential utility in mitigating oxidative stress and inducing Nrf2 transcriptional expression.

**FIGURE 8:**
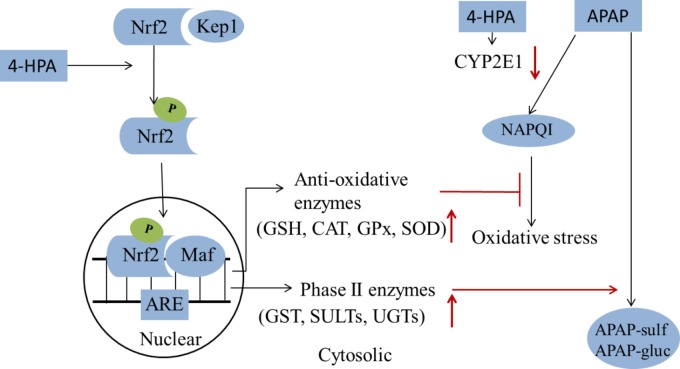
A summary of 4-HPA’s effects on the APAP-induced injury. 4-HPA may stimulate the Nrf2/Keap1 into phosphorylated Nrf2 by MAPK, P13, ERK or JNK. In the nuclear, the phosphorylated Nrf2 combines with one of small Maf proteins and binds to the antioxidant response element (ARE) in the upstream promoter region of many antioxidative genes and phase II enzymes, and initiates their transcription. 4-HPA promotes the APAP transformation into a non-toxic form. 4-HPA increased Nrf-2 translocation to the nucleus and enhanced the activity of phase II enzymes and anti-oxidant enzymes, and could thereby ameliorate APAP-induced liver injury. It can inhibit the activity of CYP2E1, alleviate the oxidative stress of the liver, and counter the liver toxicity induced by APAP. The red arrows marks indicated that the results of 4-HPA treatments.

In this study, the ability of 4-HPA in reducing liver dysfunction was evaluated and compared to the NAC under APAP-induced ALF. 4-HPA was more effective at a lower dose than NAC. It is worthy to mention that APAP hepatotoxicity was enhanced following pretreatment with 4-HPA for 3 days at a dose of 50 mg/kg and 100 mg/kg, in our preliminary experiments. The sizes of doses of polyphenols determine the primary site of metabolism. Particularly, large doses are metabolized primarily in the liver (Bell et al., 2000). Moreover, different concentrations of phenolic acids could be involved in cellular functions and mitochondrial failure (Li et al., 2004; Fedotcheva et al., 2008). The concentration of endogenous circulating p-hydroxyphenylacetate in rat plasma is considerably higher, 410 ng/mL or 2.7 mol/L (Mani et al., 2003). The data of the present study considered that 4-HPA can prevent APAP induced liver injury through two pathways, the first way is 4-HPA down-regulated the expression of CYP2E1, which decreased plasma concentration of APAP into NAPQI; the second way is 4-HPA up-regulated the expression of detoxiciation pathway, it can stimulate Nrf2 translocation from cytoplasm into mitochondria, activate Nrf2 and thus increased the activity of antioxidant enzyme (SOD, GSH, CAT, GPx) and Phase II enzymes (GST, SULTs, and UGTs), increased expression of antioxidant genes clear APAP metabolite ROS and the increased expression of Phase II enzymes accelerated metabolism APAP in a non-toxic way, which decreased plasma concentration of APAP and accelerated APAP harmless metabolism (**Figure 8**). In this study, a pretreatment protocol was used to evaluate preventive properties; however, the curative action is not guaranteed by the article; the therapeutic effect of drug liver injury by 4-HPA should be further researched. In summary, 4-HPA, as a microbial metabolite of polyphenol, has a beneficial influence on the detoxification of APAP and is associated with the inhibited activity of CYP2E1 and the up-regulation of antioxidant enzymes and phase II enzymes via Nrf2 activation. 4-HPA could be a promising strategy for preventing drug-induced hepatotoxicity.

## Ethics Statement

The experiment was conducted using Kunming male mice (6 weeks old and 20–25 g), which were purchased from the Experimental Animal Center of the Fourth Military Medical University (Xi’an, China). The mice (8 per group) were fed a standard rodent diet and allowed free access to water. Conditions were kept at room temperature with 12-hour light–dark cycles. Mice were acclimatized to the laboratory environment one week before use. All animal experimental protocols were reviewed and approved by the Ethics Committee of Xinjiang Agricultural University for the Use of Laboratory Animals.

## Author Contributions

HZ and ZJ designed research, performed research, analyzed data, and wrote the paper. XC performed animal research. HX designed research and analyzed data. WY performed animal research. XZ designed research and organized the discussion.

## Conflict of Interest Statement

The authors declare that the research was conducted in the absence of any commercial or financial relationships that could be construed as a potential conflict of interest.
