# Supplementary material for: 4-Hydroxyphenylacetic Acid Prevents Acute APAP-Induced Liver Injury by Increasing Phase II and Antioxidant Enzymes in Mice
Source: Front Pharmacol. 2018 Jun 19;9:653. doi: 10.3389/fphar.2018.00653 (PMC6020787; doi:10.3389/fphar.2018.00653)
Supplement: Supplementary file 1 [file Table_1.docx]

**1. Docking results for Nrf2**

Molecular docking assumes that a compound is absorbed perfectly and interacted with the receptor. The lowest docking energy indicates the most significant interaction between ligand and receptor. The ligands 4-hydroxyphenylacetic acid and N-acetylcysteine (standard drug) were tested on Nrf2.

**Table. 1 Interaction profiles of the potential compounds on Nrf2 (Nuclear factor erythroid**

**2-related factor)**

| **S.No** | **Compound** | **Energy (Kcal/mol)** | **Van Der Walls** | **H-Bond** | **Electrostatic** |
| --- | --- | --- | --- | --- | --- |
| 1 | 4- Hydroxyphenylacetic acid | -59.6164 | -38.2253 | -21.3911 | 0 |
| 2 | 3,4- Dihydroxyphenylacetic acid | -67.1659 | -47.7058 | -19.4601 | 0 |
| 3 | N-acetylcysteine (Standard Drug) | -54.2193 | -48.0667 | -6.15267 | 0 |

The analysis of docking result needs various parameters of identification to describe the ligand interaction. iGemDock identifies various bond energies, such as hydrogen bond (H-Bond), Van Der Walls (VDW) interaction, and electrostatic (Dock Energy= H-Bond Energy + VDW Energy + Electrostatic Energy), which are the interactions that occur between ligand and receptor. The H-Bond interaction in ligand is related to the interaction of the hydrophilic group or the presence of atom with lone pair electron, while the VDW interaction is related to lipophilic groups such as aromatic ring, or methyl group. The docking energy was the combination of van der walls energy, hydrogen bond, and electrostatic that was the result of the interaction between ligands and macromolecules showed in Table 1.

4-Hydroxyphenylacetic acid docking scores were par with 3,4-Dihydroxyphenylacetic acid. It’s comparatively higher than the standard drug N-acetylcysteine. Which indicates the most comfortable position of ligand in a receptor, compared to both their docking energy against the Nrf2, the results may indicate that 4- hydroxyphenylacetic acid are potential to be developed as HCC therapy.

**2. Docking results for CYP2E1**

**Table. 2 Interaction profiles of the potential compounds on CYP2E1**

**(Nuclear factor erythroid 2-related factor)**

| **S.no** | **Ligand** | **Docking score** | **H-Bond interaction** | **π – π interaction** |
| --- | --- | --- | --- | --- |
| 1 | 4 Hydroxy phenylaceticacid | -8.782 | ARG 126, ARG 435, TRP 122, ARG 100 | - |
| 2 | 3,4-Dihydroxyphenylacetic acid | -6.397 | THR 304, THR 303 | **-** |

**4-Hydroxyphenylacetic acid interaction with CYP2E1**


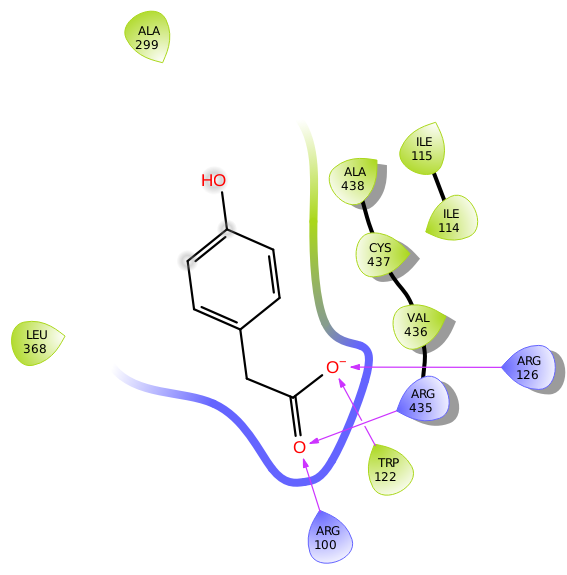


**S. Figure 1:** 4-Hydroxyphenylacetic acid interaction with CYP2E1

**3,4-Dihydroxyphenylacetic acid interaction with CYP2E1**


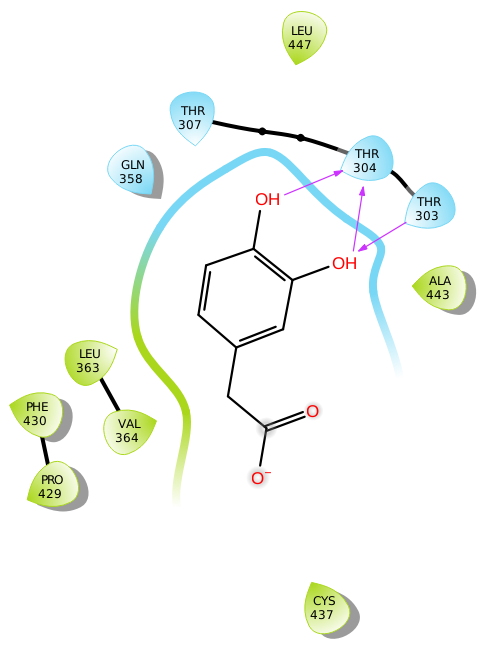


**S. Figure 2:** 3,4-Dihydroxyphenylacetic acid interaction with CYP2E1

The results show that 3,4-HPA and 4-HPA has different binding sites on CYP2E1 and 3,4-HPA were docked with CYP2E1 in hydroxyl group (Figure 1) and 4-HPA docked with CYP2E1 in carboxyl group (Figure 2). The interaction of 4-HPA was comparatively higher than the 3,4- HPA. In the present study was suggestion that 4-HPA will active natural compound to use in hepatoprotective research.
